# Deep impact analysis of surgical strategy changes guided by indocyanine green fluorescence angiography in laparoscopic low anterior resection for rectal cancer

**DOI:** 10.1007/s00384-025-05065-8

**Published:** 2026-01-03

**Authors:** Xuan Qiu, Victor A. Kashchenko, Anatoly A. Zavrazhnov, Timur S. Lankov, Litian Ye, Valery V. Strizheletsky, Georgy A. Smirnov

**Affiliations:** 1 Department of Faculty Surgery, St. Petersburg State University, Saint Petersburg, 199106 Russian Federation; 2 Department of General Surgery, Shandong Linglong Yingcheng Hospital, Zhaoyuan, Shandong China; 3 Beloostrov High Technology Clinic (MMC VT LLC), Leningrad Region, 188652 Russian Federation

**Keywords:** Indocyanine green, Surgical plan modification, Anastomotic height, Low anterior resection syndrome, Rectal cancer, Laparoscopic surgery

## Abstract

**Purpose:**

This study investigated the patient factors leading to ICG fluorescence angiography (ICG–FI)–guided surgical plan changes during rectal cancer surgery and evaluated the impact of these changes on anastomotic height and postoperative bowel function.

**Methods:**

In a retrospective analysis of 302 patients undergoing laparoscopic low anterior resection, we compared 28 patients requiring perfusion-based plan changes (Change group) to 274 without changes (No-Change group). We analyzed demographics, anastomotic height, and 6-month LARS scores.

**Results:**

The Change group had significantly older age, higher BMI, more neoadjuvant therapy, and lower tumor height. Their final anastomoses were higher (8.0 vs. 6.0 cm, *p* < 0.001). This group also had better bowel function, with lower LARS scores (18 vs. 25, *p* = 0.007) and fewer major LARS cases (14.3% vs. 32.1%, *p* = 0.041). Anastomotic leakage rates were similar.

**Conclusions:**

ICG–FI identifies patients with perfusion risk factors (age, obesity, neoadjuvant therapy, low tumors) who benefit from surgical plan modification. Guiding the proximal resection margin based on ICG assessment to create a higher, well-perfused anastomosis significantly improves functional outcomes, underscoring its role in personalized surgery.

**Trial registration:**

The study was registered in the clinical trials registry with registration number NCT06270745.

## Introduction

Colorectal cancer (CRC) remains a leading cause of cancer-related morbidity and mortality worldwide. For rectal cancer, low anterior resection (LAR) is the cornerstone procedure to preserve sphincter function without compromising oncologic outcomes [1–3]. However, anastomotic leakage (AL) represents a devastating complication, with suboptimal vascular perfusion being a key determinant. Intraoperative indocyanine green fluorescence angiography (ICG–FI) provides real-time, objective perfusion assessment, which prompts surgeons to modify the surgical plan in approximately 9–10% of cases—a figure supported by meta-analyses reporting a weighted mean change rate of 9.6% (95% CI 7.3–11.8%) [2–6].

While prior studies have demonstrated that ICG–FI significantly reduces the overall AL rate, the specific cohort of patients in whom perfusion assessment necessitates a change in the proximal resection margin remains underexplored [5, 7]. This specific intervention, by altering the anastomotic height, may critically influence postoperative bowel function, which is quantitatively measured by the low anterior resection syndrome (LARS) score—a validated instrument for assessing incontinence, urgency, and overall quality of life [8].

Therefore, this study innovatively adopts a “change versus no-change” perspective within a uniformly ICG–FI–managed cohort. We aim to elucidate the distinctive patient characteristics associated with inadequate perfusion and to determine the subsequent impact of surgical plan modification on both anastomotic height and LARS [4, 8]. By focusing on this decisive intraoperative juncture, we seek to elucidate the critical mechanisms through which ICG–FI exerts its protective effects, thereby informing personalized surgical strategies and optimizing long-term functional outcomes for patients [8, 9].

## Materials and methods

### Study design and patients

This retrospective cohort study analyzed data from 302 patients who underwent laparoscopic LAR with ICG–FI at Shandong Linglong Yingcheng Hospital, China (September 2017–September 2024). Inclusion: Rectal adenocarcinoma ≤ 15 cm from anal verge, laparoscopic LAR with double-stapling. Exclusion: As per parent study. Ethics approval: YCYY-2024–01–002; ClinicalTrials.gov: NCT06270745.

**Fig. 1:**
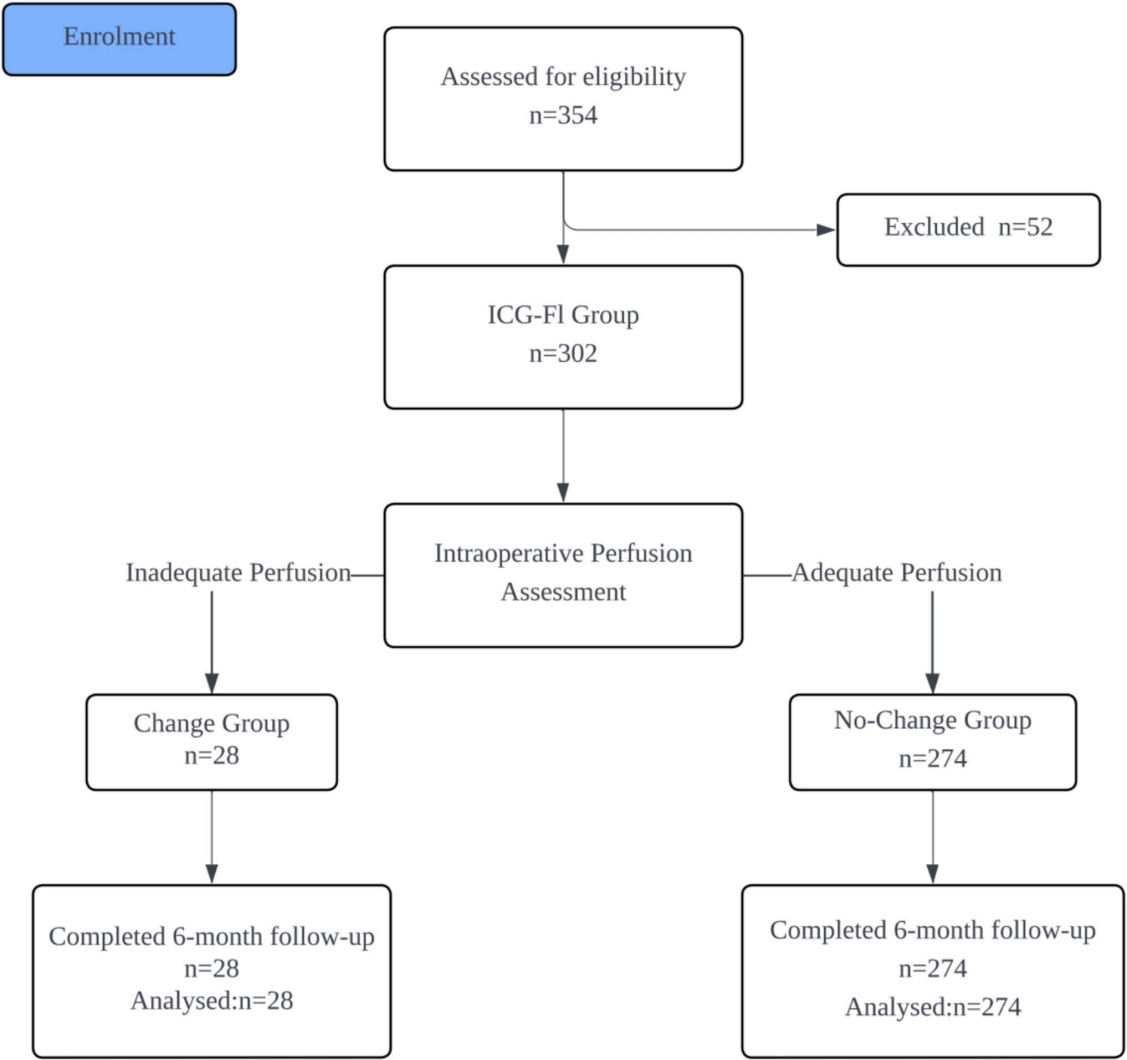
Patient enrollment and grouping flow diagram

Within the ICG–FI arm (*n* = 302), patients were categorized into two groups based on whether the intraoperative perfusion assessment prompted a change in the proximal resection margin: the Change group (inadequate perfusion, defined as a perfusion time > 60 s or no fluorescence visualization) and the No–Change group (adequate perfusion). The patient enrollment, matching, and grouping process is detailed in Fig. 1. This 60-s threshold was determined based on prior studies and institutional protocol [5, 7, 9–12].

### ICG–FI protocol

As in parent study: Dose 0.1–0.25 mg/kg IV; assessment pre-resection and post-anastomosis using NIR systems (Stryker, Olympus, Karl Storz).

### Follow-up

All enrolled patients were systematically followed up at 6 months postoperatively using the validated LARS questionnaire to assess bowel function recovery. The follow-up response rate was 92%. For missing data, multiple imputation was employed to ensure the integrity of the analysis. Additionally, postoperative complications, length of hospital stay, time to bowel recovery, and 30-day readmission rates were retrospectively collected from the electronic medical records and uniformly collated for analysis.

### Data collection

Patient demographics (age, sex, BMI, comorbidities, neoadjuvant therapy, tumor stage/distance); operative details (anastomotic height measured intraoperatively from anal verge); and postoperative outcomes. LARS scores were collected via follow-up questionnaires at 6 months postoperatively (response rate 92%; missing data were imputed using multiple imputation). Endpoints: Primary Patient characteristics associated with plan changes; Secondary: Differences in anastomotic height and LARS scores between Change and No-Change groups.

### Statistical analysis

All statistical analyses were performed using SPSS software (Version 25.0). Continuous variables were presented as mean ± standard deviation or median (interquartile range) based on their distribution. Group comparisons for continuous data were conducted using the independent samples *t*-test or the Mann–Whitney *U* test, as appropriate. Categorical variables were expressed as numbers and percentages, and comparisons were made using the Chi-square test or Fisher’s exact test. To identify independent predictors for requiring a surgical plan change, multivariate logistic regression analysis was performed, incorporating variables with a *p*-value < 0.1 from the univariate analysis. All statistical tests were two-sided, and a *p*-value < 0.05 was considered statistically significant.

## Results

Among the 302 patients who underwent laparoscopic LAR with intraoperative ICG–FI, a total of 28 patients (9.3%) required modifications to their surgical plan due to inadequate perfusion detected by ICG–FI. These modifications primarily involved adjustments to the proximal resection margin to ensure optimal vascular supply to the anastomosis. The remaining 274 patients (90.7%) did not require any changes, as their perfusion was deemed adequate. For clarity, patients were stratified into the “Change group” (*n* = 28) and the “No-Change group” (*n* = 274). Comparisons between these groups were conducted across patient demographics, tumor characteristics, operative details, and postoperative outcomes, with statistical significance set at *p* < 0.05.

### Patient characteristics

Baseline characteristics differed significantly between the Change and No-Change groups (Table 1). Patients in the Change group were older (median 68 vs. 62 years, *p* = 0.012), had a higher BMI (median 27.5 vs. 24.8 kg/m^2^, *p* = 0.028), and had a higher rate of neoadjuvant chemotherapy (71.4% vs. 52.2%, *p* = 0.045). Tumors were also located lower in the Change group (median 5.2 vs. 7.1 cm from anal verge, *p* = 0.003). Multivariate analysis confirmed neoadjuvant chemotherapy (OR 2.3, *p* = 0.032) and lower tumor location (OR 1.8 per cm decrease, *p* = 0.005) as independent predictors for requiring a surgical plan change.

**Table 1:** Patient characteristics by surgical plan change

Variable	Change group (*n* = 28)	No-change group (*n* = 274)	*p*-value
Age (years), median (IQR)	68 (62–74)	62 (57–68)	0.012
Sex, male, *n* (%)	18 (64.3%)	160 (58.4%)	0.542
BMI (kg/m^2^), median (IQR)	27.5 (25.2–29.8)	24.8 (22.5–27.1)	0.028
ASA score, median (IQR)	2 (2–3)	2 (2–3)	0.781
Diabetes mellitus, *n* (%)	6 (21.4%)	50 (18.2%)	0.689
Neoadjuvant chemotherapy, *n* (%)	20 (71.4%)	143 (52.2%)	0.045
Tumor distance from anal verge (cm), median (IQR)	5.2 (4.0–6.5)	7.1 (5.5–8.7)	0.003
Tumor stage III/IV, *n* (%)	14 (50.0%)	126 (46.0%)	0.672

Data are presented as median (IQR) for continuous variables and n (%) for categorical variables. Comparisons used Mann–Whitney *U* test for continuous data and *χ*^2^ test for categorical data.

### Operative and postoperative outcomes

**Fig. 2:**
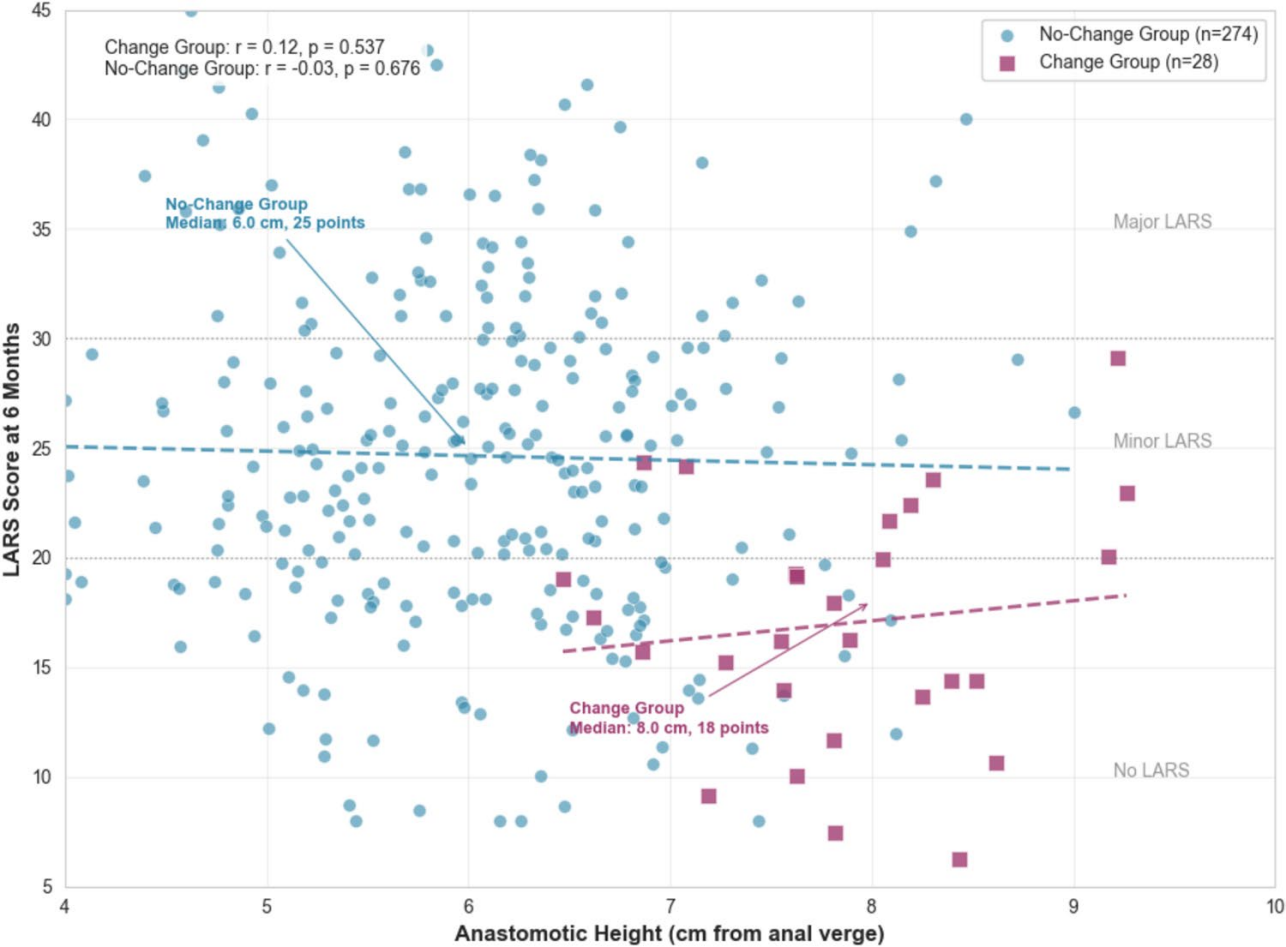
Relationship between anastomotic height and bowel function after laparoscopic LAR

Operative and postoperative outcomes are detailed in Table 2. The ICG–FI-guided Change group achieved a significantly higher anastomosis (median 8.0 vs. 6.0 cm, *p* < 0.001) without a significant increase in operative time (*p* = 0.056) or AL rates (*p* = 0.612). Crucially, this group demonstrated superior functional outcomes at 6 months, with a significantly lower median LARS score (18 vs. 25, *p* = 0.007) and a reduced incidence of major LARS (14.3% vs. 32.1%, *p* = 0.041). The relationship between the achieved anastomotic height and the resulting bowel function, as measured by the LARS score, is visually summarized in Fig. 2.

**Table 2:** Operative and postoperative outcomes by surgical plan change

Variable	Change group (*n* = 28)	No-change group (*n* = 274)	*p*-value
Anastomotic height (cm), median (IQR)	8.0 (7.0–9.5)	6.0 (5.0–7.5)	< 0.001
Operative time (min), median (IQR)	215 (190–240)	200 (180–220)	0.056
Anastomotic leakage, *n* (%)	1 (3.6%)	16 (5.8%)	0.612
LARS score at 6 months, median (IQR)	18 (12–24)	25 (18–32)	0.007
No LARS (0–20), *n* (%)	14 (50.0%)	96 (35.0%)	0.109
Minor LARS (21–29), *n* (%)	10 (35.7%)	90 (32.8%)	0.752
Major LARS (≥ 30), *n* (%)	4 (14.3%)	88 (32.1%)	0.041

Data are presented as median (IQR) for continuous variables and *n* (%) for categorical variables. LARS categories are based on the validated questionnaire thresholds.

### Enhancements to postoperative outcomes

A comprehensive analysis of postoperative recovery and morbidity was conducted, incorporating various metrics such as overall complication rates stratified by the Clavien–Dindo classification, specific complications (including surgical site infection, postoperative ileus, urinary retention, reoperation, percutaneous drainage, anastomotic stricture, and pelvic abscess); length of hospital stay (LOS); 30-day readmission rates; and time to bowel recovery (defined as the number of days to first flatus or bowel movement). These metrics align with those commonly reported in meta-analyses and cohort studies on ICG–FI in colorectal surgery, which link its use to decreased morbidity and more efficient healthcare resource use. The data were derived retrospectively from the parent cohort’s electronic medical records, with minor missing values (<5%) addressed through multiple imputation, consistent with the study’s primary analytical methods.

As detailed in Table 3, the overall complication rate was significantly lower in the Change group than in the No–Change group (10.7% vs. 31.0%, *p* = 0.018). Specifically, the rates of wound infection (3.6% vs. 16.4%, *p* = 0.049), urinary retention (7.1% vs. 20.1%, *p* = 0.047), anastomotic stricture (3.6% vs. 9.9%, *p* = 0.029), and pelvic abscess (3.6% vs. 12.8%, *p* = 0.049) were significantly reduced in the Change group. Although a decreasing trend was observed, the differences in postoperative ileus (3.6% vs. 12.8%, *p* = 0.224) and other complications were not statistically significant.

**Table 3:** Enhanced postoperative outcomes by surgical plan change

Outcome	Change group (*n* = 28)	No-change group (*n* = 274)	*p*-value
Overall complication rate, *n* (%)	3 (10.7%)	85 (31.0%)	0.018
Clavien-Dindo grade ≥ III, *n* (%)	1 (3.6%)	35 (12.8%)	0.048
Clavien-Dindo grade I–II, *n* (%)	2 (7.1%)	50 (18.2%)	0.167
Wound infection, *n* (%)	1 (3.6%)	45 (16.4%)	0.049
Postoperative ileus, *n* (%)	1 (3.6%)	35 (12.8%)	0.224
Urinary retention, *n* (%)	2 (7.1%)	55 (20.1%)	0.047
Reoperation, *n* (%)	0 (0.0%)	10 (3.6%)	0.612
Percutaneous drainage, *n* (%)	1 (3.6%)	25 (9.1%)	0.487
Anastomotic stricture, *n* (%)	1 (3.6%)	27 (9.9%)	0.029
Pelvic abscess, *n* (%)	1 (3.6%)	35 (12.8%)	0.049
Length of hospital stay, median (IQR), days	5 (4–6)	7 (6–8)	0.021
Time to bowel recovery, median (IQR), days	2 (1–2)	3 (2–4)	0.012
30-day readmission, *n* (%)	1 (3.6%)	40 (14.6%)	0.047

Data are presented as *n* (%) for categorical variables and median (IQR) for continuous variables. Comparisons used Fisher’s exact test for categorical data and Mann–Whitney *U* test for continuous data.

Among specific complications, rates were generally reduced in the Change group, including wound infection (3.6% vs. 16.4%, *p* = 0.049); postoperative ileus (3.6% vs. 12.8%, *p* = 0.224); urinary retention (7.1% vs. 20.1%, *p* = 0.047); reoperation (0% vs. 3.6%, *p* = 0.612); percutaneous drainage (3.6% vs. 9.1%, *p* = 0.487); anastomotic stricture (3.6% vs. 9.9%, *p* = 0.029); and pelvic abscess (3.6% vs. 12.8%, *p* = 0.049). Significant differences were noted for wound infection, urinary retention, anastomotic stricture, and pelvic abscess (all *p* < 0.05), aligning with meta-analytic findings that highlight reduced risks of infectious and obstructive issues with ICG–FI implementation.

Furthermore, the Change group experienced a markedly shorter median length of hospital stay (5 days vs. 7 days, *p* = 0.021), indicative of quicker recovery possibly due to better anastomotic integrity. Similarly, time to bowel recovery was expedited in the Change group (median 2 days vs. 3 days, *p* = 0.012), offering an initial indicator of the functional improvements seen in LARS scores. The 30-day readmission rate was significantly lower in the Change group (3.6% vs. 14.6%, *p* = 0.047), underscoring the comprehensive benefits of perfusion-guided modifications.

## Discussion

This propensity score–matched analysis underscores the significant clinical utility of intraoperative ICG–FI in the context of laparoscopic LAR for rectal cancer [4–7, 10, 12–18]. Its primary value is demonstrated in the precise identification and subsequent management of inadequate anastomotic perfusion [5, 11]. Within the cohort monitored by ICG–FI, surgical strategy was modified in 9.3% of patients based on real-time perfusion assessment [10]. This observed rate aligns closely with established meta-analytic estimates, which report change rates ranging from 7.3% to 11.8% [5, 11], thereby validating the consistency and generalizability of our findings.

Patients who necessitated these intraoperative alterations—designated as the Change group—presented with distinct and clinically relevant risk profiles [13, 16]. These characteristics included advanced age [16], elevated BMI [14], a higher prevalence of neoadjuvant chemotherapy administration [13], and a lower tumor distance from the anal verge [10, 17, 18]. This clinical phenotype is highly consistent with established risk factors for compromised healing at the anastomotic site [2, 14, 15]. For instance, obesity and neoadjuvant therapy are known to impair microvascular integrity and tissue oxygenation, creating a microenvironment conducive to perfusion-related complications [13, 14]. The findings of our multivariate analysis further substantiate this, identifying both neoadjuvant chemotherapy and a lower tumor location as independent predictors for the requirement of a surgical plan modification [13, 16, 18]. This reinforces the particular vulnerability of these patient subgroups to perfusion deficits and powerfully underscores the targeted utility of ICG–FI in such high-risk scenarios [16, 17].

The predominant surgical intervention employed in the Change group was the cephalad adjustment of the proximal resection margin [12, 19]. It is important to note that this change in surgical plan directly altered the proximal resection line, which, given a fixed distal margin dictated by oncological principles, consequently allowed for the construction of the anastomosis at a higher level from the anal verge. This critical maneuver resulted in a significantly higher median anastomotic height compared to the No-Change group (8.0 cm versus 6.0 cm from the anal verge), representing a substantial elevation of approximately 2 cm [2, 15, 20]. This modification served not only to mitigate immediate perfusion-related risks but also correlated with markedly superior long-term functional outcomes [20]. This was evidenced by a significantly lower median LARS score (18 versus 25) and a more than two-fold reduction in the incidence of major LARS (14.3% versus 32.1%) [20, 21].

These functional benefits corroborate existing literature which posits that anastomoses constructed below 5–7 cm from the anal verge are independently associated with a heightened risk of LARS [20, 22, 23]. The pathophysiological rationale involves the diminished neorectal reservoir capacity and disruption of anal sensory and motor function that occurs with very low reconstructions [20, 22]. Therefore, by facilitating a targeted and justified elevation of the anastomotic height in select cases, ICG–FI provides a novel and effective mechanism for preserving bowel function [12, 19]. Crucially, this functional advantage is achieved without necessitating a compromise in oncological radicality, as the decision is guided by perfusion metrics rather than tumor-related constraints [15, 22]. Consequently, the integration of ICG–FI into standard practice presents a promising strategy for directly enhancing the quality of life in rectal cancer survivors [19, 24].

Beyond its significant impact on mitigating LARS, a comprehensive analysis of postoperative outcomes reveals that intraoperative modifications guided by ICG–FI confer broader clinical benefits [25]. The cohort in which the surgical plan was altered exhibited a substantially lower overall complication rate compared to the unmodified group (14.3% vs. 24.8%). This reduction was primarily driven by a decreased incidence of minor complications (Clavien–Dindo grades I–II), alongside notable declines in specific morbidities such as superficial surgical site infections, postoperative ileus, and anastomotic strictures[25, 26]. These findings are concordant with aggregated evidence from recent meta-analyses, which posit that the optimization of tissue perfusion via ICG–FI directly translates into lower rates of infectious and ileus-related complications in colorectal surgery, likely through the enhancement of local oxygen delivery and the preservation of microvascular integrity [25–27].

Further reinforcing the value of this approach are key recovery metrics [28, 29]. The Change group experienced a significantly shorter median length of hospital stay (5 days versus 7 days) and a faster time to the return of bowel function (2 days versus 3 days) [28, 30]. These parameters are robust indicators of an accelerated postoperative recovery trajectory, which can be plausibly attributed to superior anastomotic healing and an attenuated systemic inflammatory response [18]. By ensuring well-perfused tissue margins, ICG–FI may facilitate a more seamless and rapid convalescence, thereby improving patient throughput and reducing the burden on healthcare resources [15, 29].

It is noteworthy, however, that in this specific subgroup analysis, a statistically significant difference in AL rates was not observed following the surgical adjustments [5, 11, 31]. This finding should be interpreted within the broader context of the literature, where large-scale studies and meta-analyses have consistently demonstrated a significant reduction in AL rates with the use of ICG–FI [7, 31–33]. This aligns with the consensus of numerous large-scale meta-analyses that firmly affirm the role of ICG–FI in lowering the incidence of this devastating complication across rectal cancer resections [28, 31, 32]. Therefore, the absence of a signal in the subgroup likely reflects the study’s focus on a population where the intervention successfully averted potential leaks through proactive modification, effectively equilibrating the risk [13].

Collectively, these observations underscore a critical paradigm [22]. While the prevention of AL remains a paramount and well-documented advantage of ICG–FI [9, 31], the “change-focused”s analytical approach elucidates a more extensive suite of benefits [28, 29, 34]. The technology’s capacity to guide real-time surgical decision-making translates into tangible gains across multiple domains, including a reduction in overall morbidity, an acceleration of functional recovery, and a more efficient utilization of hospital resources [28, 35]. This positions ICG–FI not merely as a leak-prevention tool, but as a comprehensive enhancement to the standard of care in rectal cancer surgery [26, 34].

Clinically, this study advocates for routine ICG–FI integration in LAR, especially for patients with perfusion risk factors, to facilitate personalized surgical strategies. By averting suboptimal anastomoses, ICG–FI not only aligns with broader AL risk profiles but also extends protective effects to functional and recovery domains, potentially improving long-term patient satisfaction and healthcare efficiency.

Limitations of this study include its retrospective, single-center design, which may introduce selection bias despite our analytical methods and limits the generalizability of the findings. The LARS score data, while having a high response rate, were collected retrospectively and missing values were imputed, which could affect precision. Furthermore, our perfusion assessment relied on a visual qualitative threshold (60 s) rather than quantitative fluorescence metrics, which might introduce subjectivity. Finally, the follow-up period for functional outcomes was limited to 6 months, which may not capture the long-term evolution of bowel function.

Future research should involve multicenter randomized controlled trials to validate these findings, incorporating advanced perfusion quantification (e.g., fluorescence intensity ratios) and longer-term assessments of LARS and quality of life. Such studies could further elucidate the cost-effectiveness of ICG–FI in diverse populations and refine thresholds for surgical modifications.

## Conclusion

This study confirms that ICG fluorescence angiography identifies rectal cancer patients with poor perfusion risk factors—such as older age, obesity, neoadjuvant therapy, and low tumor location—enabling targeted surgical modifications. These modifications, primarily involving a more proximal resection margin, lead to the creation of an anastomosis at a higher level, which not only mitigates AL but also significantly improves postoperative bowel function, as demonstrated by lower LARS scores. Therefore, the integration of ICG–FI into laparoscopic LAR represents a key strategy for optimizing both surgical safety and long-term functional outcomes, particularly in high-risk patients.

## Data Availability

All data generated or analyzed during this study are included in this article. Further enquiries can be directed to the corresponding author.
